# Ulvan, carrageenan, and fucoidan: comparative immunomodulatory mechanisms and future directions in major algal sulfated polysaccharides

**DOI:** 10.3389/fimmu.2026.1949951

**Published:** 2026-10-12

**Authors:** Shuang Zhao, Zhangyi Xia, Jinlin Liu, Meijing Zhang, Maozi Lin, Guangmao Ding, Longchen Tang, Fang Su, Jingyi Sun, Chao Zhao

**Affiliations:** 1 Fujian Provincial Key Laboratory of Coastal Basin Environment, Fujian Polytechnic Normal University, Fuqing, China; 2 Ocean College, Fujian Polytechnic Normal University, Fuqing, China; 3 State Key Laboratory of Marine Environmental Science, College of Ocean and Earth Sciences, Xiamen University, Xiamen, China; 4 Key Laboratory of Marine Ecological Monitoring and Restoration Technologies, East China Sea Ecological Center, Ministry of Natural Resources, Shanghai, China; 5 Project Management Office of China National Scientific Seafloor Observatory, Tongji University, Shanghai, China; 6 College of Life Sciences, Fujian Agriculture and Forestry University, Fuzhou, China; 7 Hebei Engineering Research Center for Ecological Restoration of Seaward Rivers and Coastal Waters, Hebei University of Environmental Engineering, Qinhuangdao, China; 8 College of Marine Sciences, Fujian Agriculture and Forestry University, Fuzhou, China

**Keywords:** carrageenan, cGAS-STING signaling pathway, fucoidan, innate immunity, NLRP3 inflammasome, receptor recognition, structure-activity relationship, ulvan

## Abstract

Sulfated polysaccharides derived from marine algae have attracted considerable interest in innate immunity research due to their structural diversity and pronounced immunomodulatory activities. Ulvan, carrageenan, and fucoidan, derived from green, red, and brown algae, respectively, represent three paradigmatic classes of algal sulfated polysaccharides. This review establishes a four-tier analytical framework, namely “structural features, receptor recognition, signaling pathways, and immune outcomes”, to systematically compare their mechanistic differences in innate immune modulation. This framework provides, for the first time, a unified causal chain from molecular structure to immune effector phenotypes. Ulvan is characterized by coordinated TLR4/cGAS-STING dual pathways: it drives pro-inflammatory responses via the TLR4-MyD88-NF-κB axis while inducing type I interferon production through the cGAS-STING-TBK1-IRF3 cascade. TBK1, as a shared downstream node of both the TLR4-TRIF and cGAS-STING pathways, may mediate signal integration and amplification, thereby conferring a distinctive advantage to ulvan in antiviral immunity. Carrageenan exhibits dual TLR4/NLRP3 activities, with κ/ι/λ subtypes displaying a functional spectrum from localized pro-inflammation to systemic immunostimulation, determined by their sulfate content and gelation properties. A critical distinction must be made between high-molecular-weight native carrageenan (>100 kDa), which is minimally absorbed in the intestine and considered safe, and low-molecular-weight degraded carrageenan (<50 kDa), which carries inflammatory and toxic risks. Fucoidan achieves immunomodulatory pleiotropy through dual TLR4/CLR recognition and concurrent engagement of multiple signaling cascades, encompassing NF-κB-driven pro-inflammation, bidirectional NLRP3 inflammasome regulation, and complement C3 activation. The antiviral effect of fucoidan via the STING pathway, potentially dependent on ROS-mediated DNA damage or endoplasmic reticulum stress, mechanistically differs from ulvan’s cGAS-STING activation. The structural basis driving this pathway selectivity resides in an inverse gradient relationship between sulfate and uronic acid contents across the three polysaccharides: sulfate content ranks as fucoidan > carrageenan > ulvan, while uronic acid content follows the opposite order. This inverse gradient serves as the first unified structural principle explaining differential receptor preferences and pathway selection among these three distinct classes of algal polysaccharides. Based on this framework, we propose differentiated polysaccharide design strategies tailored to three therapeutic objectives (pro-inflammation, antiviral defense, and immune balance), offering a roadmap for structure-guided immunomodulation using algal sulfated polysaccharides.

## 1 Introduction

Marine algae represent one of the most abundant renewable biological resources on Earth, and their cell walls and intercellular matrices are rich in structurally diverse sulfated polysaccharides (1). Unlike neutral polysaccharides derived from terrestrial plants, algal sulfated polysaccharides possess unique negative charge characteristics due to the presence of sulfate ester groups, enabling electrostatic interactions with various immune-related proteins and consequently exhibiting significant innate immunomodulatory activity (2). In recent years, with the interdisciplinary convergence of marine glycobiology and immunology, research on algal sulfated polysaccharides as natural immunomodulators has grown exponentially, and the relevant literature now covers a complete chain from chemical structure elucidation to *in vivo* efficacy evaluation (3).

Ulvan, carrageenan, and fucoidan are three representative algal sulfated polysaccharides, derived from the green algal genus *Ulva* (phylum Chlorophyta), the red algal genus *Chondrus* (phylum Rhodophyta), and the brown algal genus *Fucus* (phylum Phaeophyta), respectively. They exhibit systematic differences in monosaccharide composition, sulfate content and position, molecular weight distribution, and glycosidic linkage types, thus providing a natural research system for comparing structure-immunoactivity relationships (4). Ulvan features a backbone of rhamnose-glucuronic acid disaccharide repeating units, with a low degree of sulfation but a high content of uronic acids (5); carrageenan is based on a galactose backbone, and its three subtypes (κ, ι, and λ) exhibit markedly different gelling abilities and immunomodulatory activities due to variations in the number and position of sulfate groups (5, 6); fucoidan, with fucose as its main chain, is the most highly sulfated and structurally diverse among the three (6–8).

A central bottleneck in current research is that mechanistic studies on the immunomodulatory effects of these three polysaccharides have been conducted independently, lacking a unified comparative analytical framework. Recent work suggests that ulvan may activate the cGAS-STING/STING pathway, although direct cGAS involvement remains to be demonstrated (9); carrageenan research has long revolved around its dual role as both an inflammatory model tool and a food additive (10); while the literature on fucoidan has emphasized multi-receptor recognition and structure-activity relationships (11). This fragmented landscape gives rise to three specific scientific problems. First, the differences among the three polysaccharides in receptor preference, signaling pathway selectivity, and immunoeffector phenotypes cannot be systematically understood; second, a unified understanding of the structure–activity relationships linking chemical structural parameters (sulfate content, uronic acid content, molecular weight) to immunological activity is lacking across different polysaccharide types; and third, there is no mechanism-based rational criterion for defining the applicable boundaries of each polysaccharide in different disease contexts.

To address these gaps, we employ a four-tier analytical framework to systematically compare the innate immunomodulatory mechanisms of the three polysaccharides across four levels: sulfation patterns, pattern recognition receptor (PRR) recognition profiles, downstream signaling pathway activation preferences, and immune effector phenotypes. The theoretical innovations of this work are twofold. First, for the first time, we place three algal sulfated polysaccharides from distinct algal divisions under a unified four-tier framework for horizontal comparison, breaking through the previously fragmented research landscape. Second, we distill from the structural features of the three polysaccharides a core structure-activity principle—namely, that sulfate content and uronic acid content exhibit an inverse gradient relationship—which provides the first unified structur al explanation for the observed differences in receptor preference and pathway selection across polysaccharide types. In terms of applied innovation, based on this inverse gradient relationship, we extrapolate differentiated usage strategies and combination strategies for the three polysaccharides across three disease scenarios (viral infection, tumor immunity, and allergic diseases), thereby offering an actionable roadmap for rational polysaccharide design grounded in structural characteristics. This four-tier causal chain can be summarized as follows: structural features (sulfate groups, uronic acids, molecular weight) determine receptor recognition preferences; receptor preferences drive pathway selection; pathway combinations shape effector phenotypes; and ultimately, effector phenotypes dictate the applicable scenarios. As shown in **Figure 1**, the four levels form a causal chain from molecules to cells, providing a unified narrative structure for the mechanistic dissection of individual polysaccharides and cross-comparison in subsequent sections.

**Figure 1:**
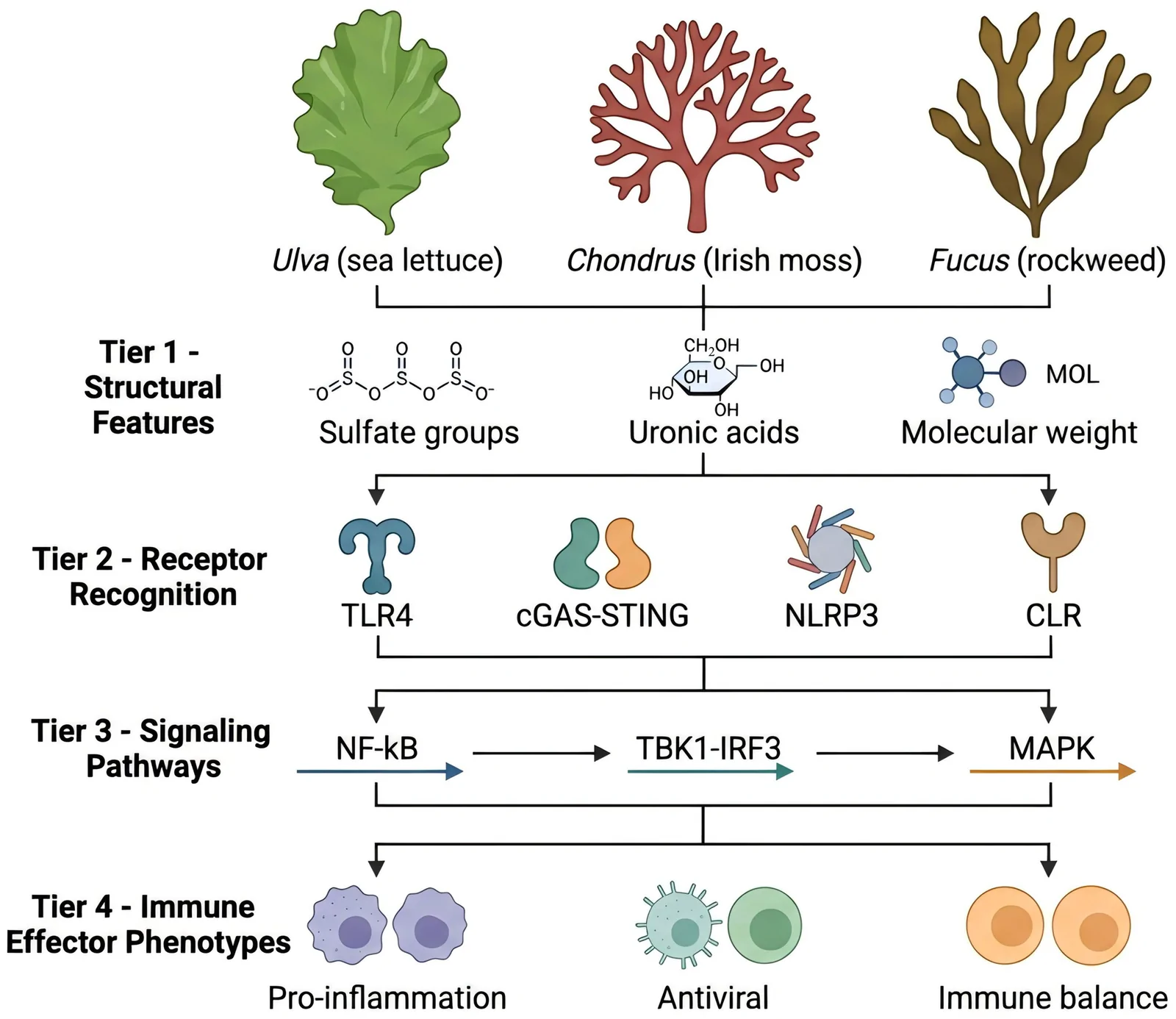
Four-tier analytical framework for the innate immunomodulatory mechanisms of three algal sulfated polysaccharides. This figure outlines the causal progression across four levels (structural features, receptor recognition, signaling pathways, and immune effector phenotypes), thereby providing a unified analytical framework for the mechanistic dissection of individual polysaccharides and their subsequent cross-comparison.

The theoretical innovations of this paper are twofold. First, for the first time, we place three algal sulfated polysaccharides from distinct origins under a unified four-tier framework for horizontal comparison, breaking through the previously fragmented research landscape. Second, we distill from the structural features of the three polysaccharides a core structure-activity principle (namely, that sulfate content and uronic acid content exhibit an inverse gradient relationship), which provides a unified structural explanation for the observed differences in receptor preference and pathway selection. In terms of applied innovation, based on this inverse gradient relationship, we extrapolate differentiated usage strategies and combination strategies for the three polysaccharides across three disease scenarios (viral infection, tumor immunity, and allergic diseases), thereby offering an actionable roadmap for rational polysaccharide design grounded in structural characteristics.

To ensure a comprehensive and systematic literature review, we searched PubMed, Web of Science, Scopus, and CNKI from inception to June 2026 using keywords including “ulvan”, “carrageenan”, “fucoidan”, “innate immunity”, “TLR4”, “NLRP3”, and “cGAS-STING”. Only peer-reviewed articles that provided adequate structural characterization of the polysaccharides were included, while crude extracts without validation were excluded. Reference lists of the retrieved articles were cross-checked to identify additional relevant studies, and data were integrated qualitatively, with structural annotation performed for each cited work.

## 2 Structural features and sources of three algal sulfated polysaccharides

The structural differences among the three polysaccharides constitute the material basis for understanding their divergent immunomodulatory activities. This section compares them across four levels (monosaccharide composition, sulfate content and position, molecular weight distribution, and solution conformation), while also addressing the effects of their respective algal sources and extraction methods on the resulting structures. A summary of key structural parameters is provided in **Table 1**.

**Table 1:** Comparison of structural features of the three algal sulfated polysaccharides.

Structural feature	Ulvan	Carrageenan (κ/ι/λ)	Fucoidan	References
Main monosaccharides	Rhamnose, glucuronic acid	D-Galactose, 3,6-anhydro-D-galactose	L-Fucose	(2)
Sulfate content (%)	8–27	15–40	5–38	(7, 12–14)
Sulfate substitution sites	C-3 of rhamnose	C-4 (κ), C-2/C-4 (ι), C-2/C-6 (λ)	C-2/C-4 of fucose	(15)
Uronic acid content (%)	6.5–19.0	Low or absent	0.52–26.88*	(12, 16–18)
Molecular weight (kDa)	10–3600	100–1000	10–950	(12, 13, 18–20)
Gelation ability	Yes	Yes (κ/ι); No (λ)	No	(21–23)
Algal source	*Ulva/Enteromorpha* (Chlorophyta)	*Chondrus*, *Eucheuma* (Rhodophyta)	*Fucus*, *Laminaria* (Phaeophyta)	(24–26)

*The upper value (26.88%) corresponds to a specific low-molecular-weight fraction (0.5 M DFPS) of *Saccharina japonica* fucoidan; most fucoidan fractions contain much lower uronic acid levels (17).

### 2.1 Ulvan: chemical structure and algal source

Ulvan is the major polysaccharide component of the cell walls of algae belonging to the green algal order Ulvales (phylum Chlorophyta), and its chemical structure features a backbone of disaccharide repeating units composed of alternating 3-sulfated rhamnose and glucuronic acid (or iduronic acid) (27). Ulvan is mainly derived from various green algae of the genus *Ulva* (including *Enteromorpha*), which are widely distributed in coastal waters worldwide and are amenable to large-scale cultivation and harvesting (28). Notably, *Ulva* spp. are also responsible for massive green tides that have been reported globally, with annual peak biomass exceeding 5 million tons in the Yellow Sea alone, and emerging evidence indicates that these blooms are regulated by dynamic host-microbiota interactions (29). Ulvan exhibits a broad molecular weight range, with molecular weights spanning from 10 to 3600 kDa (12, 13, 18), a sulfate content of 8%–27% (12, 13), and a total uronic acid content of 6.5%–19.0% (12, 18). The abundant uronic acid carboxyl groups along the molecular chains confer outstanding metal ion chelating capacity to this polysaccharide (12, 18, 30).

Extraction methods significantly affect the structure and bioactivity of ulvan (31–33). Hot water extraction yields ulvan with high molecular weight but at a low yield (31, 32); alkaline extraction tends to cause loss of sulfate groups (31), whereas acidic extraction may cleave glycosidic bonds (33). In contrast, enzymatic methods (33) and microwave/ultrasound-assisted methods (32) can improve the yield under mild conditions. Moreover, ultrasound/microwave extraction, by increasing the sulfate and uronic acid contents, exhibits stronger antioxidant and anti-skin-aging activities (32). In addition, ulvan exhibits other biological activities such as anticoagulant effects (16), and holds application potential in the food, cosmetic, and pharmaceutical fields. Studies have shown that high molecular weight ulvan (209 kDa) possesses stronger immunomodulatory activity, whereas low molecular weight (7 kDa) displays no significant activity, indicating that molecular weight is a key parameter affecting its bioactivity (34). Ulvan is unique among the three polysaccharides in simultaneously possessing high uronic acid and low sulfate content, a structural profile that directly shapes its distinctive TLR4/cGAS-STING dual-pathway signature (see Section 3).

### 2.2 Carrageenan: type diversity and molecular basis

Carrageenan is a class of linear sulfated galactans extracted from red algae, with a backbone composed of alternating D-galactose and 3,6-anhydro-D-galactose units. Depending on the differences in sulfate position and content, carrageenan is mainly classified into three types: κ-type (one sulfate group at C-4), ι-type (two sulfate groups at C-4 and C-2), and λ-type (sulfate at C-2, with partial substitution also at C-6) (15). Owing to the presence of 3,6-anhydrogalactose residues, κ- and ι-type carrageenans can form double-helicals upon cooling and further aggregate into three-dimensional gel networks (22); whereas λ-type, lacking 3,6-anhydrogalactose and having the highest sulfation degree, cannot gel and exists in solution as soluble random coils (35).

Reflecting their different degrees of sulfation (one, two, and three sulfate groups per disaccharide unit, respectively), κ-, ι-, and λ-carrageenans display three distinct physical behaviors (rigid gel, flexible gel, and non-gelling) (36). This variation in gelling ability determines their *in vivo* forms and modes of contact with immune cells, and thus exerts profound influences on their immunological activities: the rigid gel of κ-carrageenan can create a local sustained stimulation interface, and oral administration can modify peritoneal cell composition and activate the TLR4-NF-κB and Bcl10-NF-κB inflammatory pathways (37–39); λ-carrageenan, being completely soluble, diffuses rapidly and can activate monocytes in a unique manner through binding to serum IL-8, and its immunostimulatory potency increases with sulfation degree (40). Thus, the relationship between the gelling behavior and immunological activity of carrageenan is essentially determined by sulfation degree, a unifying structural parameter (36, 41).

Carrageenan is primarily obtained from various seaweeds of the phylum Rhodophyta, including the genera *Chondrus*, *Eucheuma*, and *Gigartina* (19, 42). Notably, carrageenans from different algal sources show marked differences in type composition (such as the commercially relevant κ-, ι-, and λ-types) (42), and their molecular weight ranges broadly (typically between 100 and 1000 kDa) depending on the algal source and extraction process (19). Carrageenan is unique among the three polysaccharides in that its gelation properties can be tuned through type selection, a characteristic that has direct implications for its dual role as both an inflammatory model tool and a potential immunotherapeutic agent (see Section 4).

### 2.3 Fucoidan: molecular weight, sulfation pattern, and structural heterogeneity

Fucoidan is a class of brown algal sulfated polysaccharides with a backbone composed primarily of α-L-fucose, and represents the most structurally complex among the three polysaccharides (11, 43). Its backbone typically consists of fucose residues linked by α-1,3- or alternating α-1,3- and α-1,4-glycosidic bonds, with sulfate groups predominantly at C-2 and/or C-4, and some fucoidans also contain minor monosaccharides, including glucuronic acid, galactose, and xylose (11, 43). Fucoidans from different brown algal sources display considerable structural variation; key parameters, including molecular weight (5–1000 kDa), sulfate content (5%–38%), and uronic acid content, are summarized in **Table 1**, with detailed ranges and source-specific variations provided therein. The substitution pattern of sulfate groups (for instance, C-4 sulfation in chondroitin sulfate) can markedly modulate TLR4-mediated inflammatory responses (44). Although natural fucoidans commonly bear sulfation at C-2 and C-4 (45, 46), their direct contribution to TLR4 binding requires further validation. Branching structures, such as (1→6)-β-linked side chains, have been shown to significantly enhance the recognition affinity of C-type lectin receptors like Dectin-1 for glucans (47).

The immunomodulatory activity of fucoidan depends critically on two key parameters: sulfate content and molecular weight. Higher sulfate content provides a crucial structural foundation for strong immunostimulatory activity (48–50). Fucoidan fragments with a moderate degree of polymerization (e.g., 10–50 kDa) typically exhibit potent immunomodulatory effects (49, 51). In contrast, high-molecular-weight fractions (>100 kDa) often display reduced activity owing to steric hindrance or poor membrane penetration (50), whereas excessive degradation to ultra-low molecular weights (e.g., a few kDa) may disrupt structural integrity and consequently diminish activity (48, 49). The substitution pattern of sulfate groups (for instance, C-4 sulfation in chondroitin sulfate) can markedly modulate TLR4-mediated inflammatory responses (44). With respect to extraction, hot water treatment yields native high-molecular-weight polysaccharides (52, 53), whereas acid hydrolysis and enzymatic degradation enable controlled molecular weight reduction (54). By optimizing degradation parameters (e.g., incubation time and enzyme concentration), low-molecular-weight products with defined size ranges can be generated; these fragments display markedly distinct immunomodulatory profiles (53–55), underscoring molecular weight as a critical structural determinant of bioactivity. Compared with ulvan and carrageenan, fucoidan is distinguished by high sulfate and generally low uronic acid contents, placing it at the high-sulfate pole of the sulfate/uronic acid inverse gradient (**Table 1**). This structural feature underpins its multi-pathway activation mode involving TLR4 and CLRs (see Section 5).

### 2.4 Summary of structural features of the three polysaccharides

The structural features of the three polysaccharides exhibit a progressive hierarchy of differences, as summarized in **Table 1**. Taken together, the sulfate content and uronic acid content display an inverse gradient relationship: sulfate content ranks as fucoidan > carrageenan > ulvan, whereas uronic acid content follows the opposite order, with ulvan being significantly higher than carrageenan and fucoidan (the latter two generally having low uronic acid levels, with the exception of specific low-molecular-weight fucoidan fractions that may contain elevated uronic acid, e.g., up to 26.88% in certain fractions as reported by 17). This inverse gradient serves as the structural basis for the differential receptor preferences and pathway selection elaborated in the following sections and represents the first unified structure-activity principle applicable across all three polysaccharide classes.

**Figure 2** illustrates the clear progressive differences in monosaccharide backbones, sulfate content, and substitution positions among the three polysaccharides. Ulvan features a rhamnose-glucuronic acid disaccharide repeating unit with low sulfation but high uronic acid content; the three carrageenan subtypes (κ, ι, λ) form a complementary series based on a galactose backbone and distinct sulfate positioning; fucoidan possesses a highly sulfated fucose main chain with diverse substitution patterns, making it the most structurally plastic of the three. These structural distinctions provide the molecular basis for their differential receptor recognition and signaling pathway selectivity, and the inverse gradient of sulfate versus uronic acid content is already evident at the structural level.

**Figure 2:**
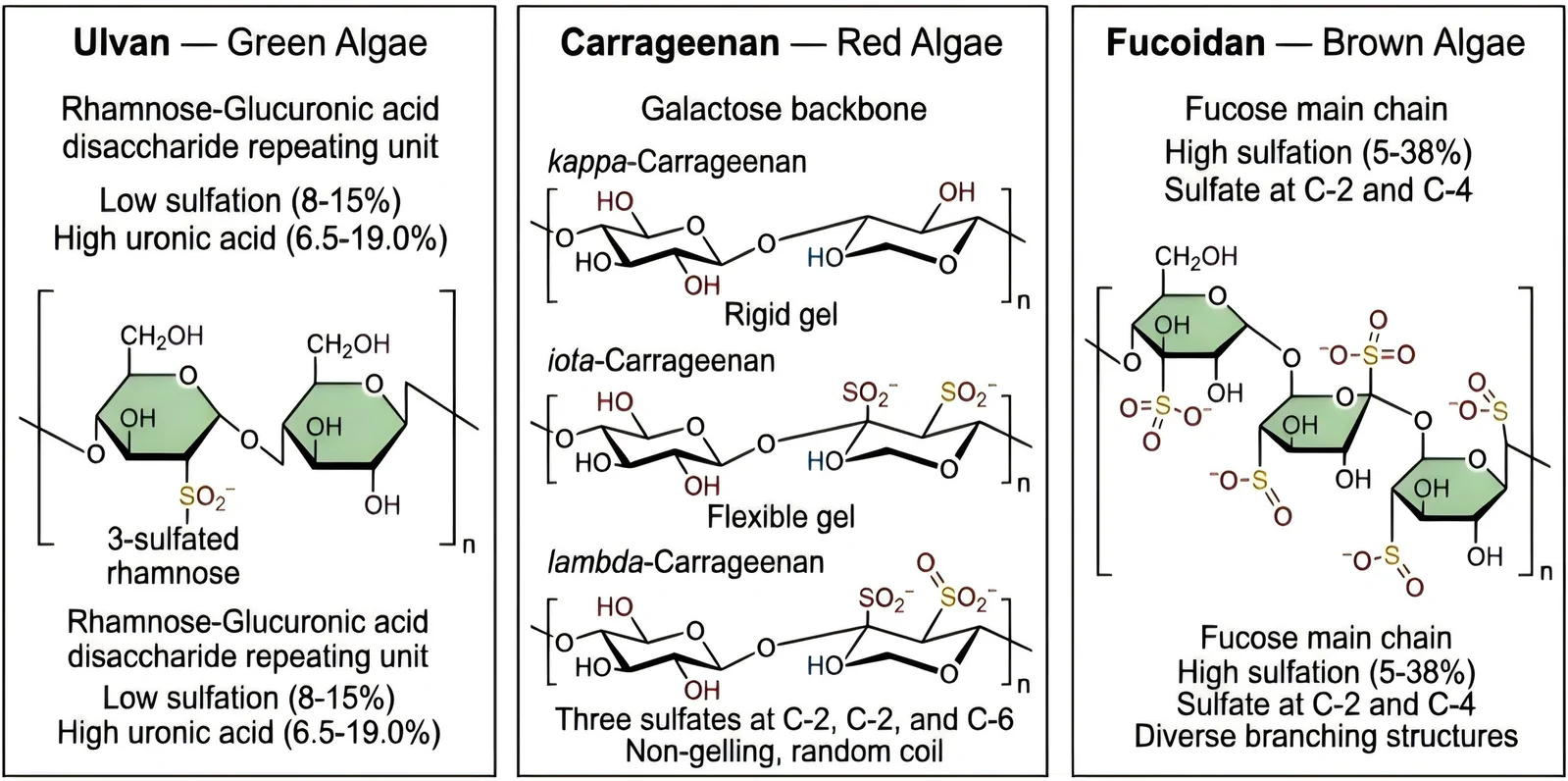
Comparison of the chemical structures of the three algal sulfated polysaccharides.

## 3 Ulvan: innate immunomodulatory mechanisms

The immunomodulatory mechanism of ulvan is characterized by the coordinated action of a TLR4-mediated pro-inflammatory pathway and a cGAS-STING-mediated antiviral pathway. Kim et al. (56) demonstrated, through antibody blockade experiments, that low-molecular-weight ulvan (approximately 1–5 kDa) activates downstream MAPK/NF-κB signaling via surface TLR2/4 to drive pro-inflammatory responses. Shah et al. (57) corroborated the regulation of the TLR4/NF-κB pathway by ulvan-type polysaccharides in a neuroinflammation model. Zhang et al. (58) found that ulvan from *Ulva pertusa* was ineffective in IFN-deficient Vero cells but significantly induced IFN-β and IFIT1 in macrophages. Their concurrent in-depth study (9) further revealed that a medium-to-low-molecular-weight ulvan fraction (PR1, 13.5 kDa) can enter cells and activate the cGAS-STING-TBK1-IRF3 axis, a mechanism independent of TLR4 signaling. This coordinated mode of surface TLR engagement and intracellular STING activation is unique among algal sulfated polysaccharides.

### 3.1 TLR4/TLR2-mediated macrophage activation

The immune activation of macrophages by ulvan begins with its recognition by cell surface pattern recognition receptors such as TLR2 and TLR4. Studies have confirmed that low-molecular-weight ulvan (LMWU, approximately 1–5 kDa) can initiate signaling through TLR2 and TLR4, activate the MAPK and NF-κB pathways, and thereby drive the transcription and release of pro-inflammatory cytokines (56). Similarly, transcriptomic analysis of ultra-low-molecular-weight ulvan oligosaccharides (OUP, 542 Da) predicted activation of multiple PRR-related signaling pathways, including the TLR family and downstream MyD88-dependent pathways, with significant upregulation of TNF-α, IL-1β, IL-6, and iNOS expression (59). Notably, in that study, the MAPK signaling pathway was downregulated rather than activated, which may be related to the specific mode of action of ultra-low-molecular-weight oligosaccharides. In addition, a crude ulvan fraction (40 kDa) was shown to induce secretion of IL-6, IL-1β, and NO in human macrophages without significant cytotoxicity; however, recognition receptors and signaling pathways were not examined in that study (60). These results indicate that molecular weight is a key structural parameter regulating the immunomodulatory activity of ulvan, with low-molecular-weight fragments (<30 kDa) exhibiting significant pro-inflammatory immunostimulatory capacity, supporting their potential development as immunoenhancers or vaccine adjuvants. This molecular-weight dependence suggests that immunologically active ulvan fragments trigger MyD88-dependent signal transduction via recognition by the TLR4/MD-2 complex, thereby activating the IKK complex, promoting IκBα phosphorylation and degradation, and nuclear translocation of NF-κB p65, ultimately initiating the transcription of pro-inflammatory cytokine genes.

Kim et al. (56) prepared low-molecular-weight ulvan (approximately 1–5 kDa) from the Korean seaweed *Ulva pertusa* and confirmed, through both *in vitro* and *in vivo* experiments, that it activates MAPK (ERK, JNK, p38) and NF-κB signaling pathways via TLR2/TLR4, while also promoting short-chain fatty acid (SCFA) production and G-protein-coupled receptor (GPCR) signaling. Multiple studies have demonstrated immunostimulatory activity for low-molecular-weight ulvan/oligosaccharides. For example, Kim et al. (56) found that low-molecular-weight ulvan (1–5 kDa) promotes TNF-α secretion by macrophages and restores *in vivo* IL-12 levels. Guevara-Torrejón et al. (60) confirmed that crude ulvan (40 kDa) upregulates CD86 expression in human macrophages, although receptor and pathway mechanisms were not examined. Wu et al. (59) demonstrated that ulvan oligosaccharides (542 Da) induce TNF-α and iNOS expression with immunoadjuvant activity, though MAPK pathways were downregulated in that study. Regarding higher-molecular-weight fractions, Kidgell et al. (34) reported that 209 kDa ulvan increased the anti-inflammatory cytokine IL-10 while also slightly upregulating IL-1β and IL-6, indicating complex immunomodulatory effects; however, receptor involvement and downstream signaling were not investigated in that study, and direct evidence for its action via “steric hindrance” affecting TLR4 or specifically inducing M2 polarization is currently lacking. Wu et al. (59) further discovered that soluble rhamnan-type ulvan oligosaccharides possess both immunostimulatory and immunoadjuvant functions, enhancing antigen-specific antibody responses, providing experimental support for the application of ulvan in vaccine adjuvants.

### 3.2 cGAS-STING pathway and antiviral immunity

The most distinctive feature of medium-to-low-molecular-weight ulvan (PR1, 13.5 kDa), which sets it apart from carrageenan and fucoidan, is its activation of the cGAS-STING signaling pathway. Zhang et al. (9) reported a significant finding: enzymatically extracted ulvan (PR1) can enter cells in an energy-dependent manner, activate the STING-TBK1-IRF3 signaling axis, promote the expression of IFN-β and interferon-stimulated genes, and thus significantly inhibit the replication of vesicular stomatitis virus (VSV). This mechanism is independent of TLR4 signaling; PR1 does not act through surface TLR4 but rather enters cells via endocytosis to directly activate the intracellular STING pathway. Furthermore, other studies have shown that ulvan possesses broad-spectrum antiviral potential. Guriec et al. (61) found that ulvan induces IFN-α release through the TLR2/4 pathway. Franco et al. (62) and Lopes et al. (63) respectively confirmed that ulvan and its chemically modified derivatives exert significant direct inhibitory effects against HSV-1 (a DNA virus), with SU1F1 achieving 100% inhibition at a concentration of 100 μg/mL. Notably, Garcia et al. (64), through screening multiple natural immune agonists, confirmed that activation of the STING pathway effectively inhibits various RNA viruses, including Zika, West Nile, Chikungunya, and SARS-CoV-2, providing important proof of concept for the broad-spectrum antiviral effects of ulvan via the STING pathway. However, whether ulvan directly upregulates cGAMP synthesis currently lacks experimental evidence and requires further investigation. This mechanism does not depend on TLR4 signaling but is achieved through ulvan entering cells and activating the intracellular STING pathway. Based on available evidence, we propose the following working hypothesis: ulvan is not a direct ligand of cGAS; rather, its mechanism may involve regulation of the cytosolic ionic microenvironment (such as Ca²^+^ signaling or the Mg²^+^/Ca²^+^ ratio) by uronic acids, thereby influencing cGAS enzymatic activity or conformational state, or indirectly transducing signals through as-yet-unidentified glycan-sensing receptors, ultimately enhancing cGAS enzymatic activity. This hypothesis awaits further validation through cGAS inhibitor intervention or gene knockout experiments.

TBK1 serves as a common downstream node of both the TLR4-TRIF pathway and the cGAS-STING pathway. Theoretically, following TLR4 activation by ulvan, the TRIF-dependent branch may converge with the cGAS-STING pathway at TBK1-IRF3, operating in parallel with the MyD88-NF-κB pro-inflammatory pathway to achieve synergistic amplification of pro-inflammatory and antiviral signals. We speculate that the TRIF-dependent branch of TLR4 activation by ulvan may participate in the integration of STING signaling; however, direct evidence for TRIF pathway activation by ulvan is currently lacking, and this hypothesis awaits further validation through TRIF gene knockout experiments. Unlike agents that directly act on viral replication, ulvan enhances the intrinsic antiviral state of host cells by activating the STING signaling pathway and inducing type I interferon (IFN-I) expression (9, 58). Because its target is the host pathway rather than viral-specific proteins, it theoretically possesses broad-spectrum antiviral activity and a lower risk of drug resistance (65). Moreover, researchers have observed significantly enhanced innate immune parameters (lysozyme activity, respiratory burst activity, complement activity, and immune gene expression) in fish fed diets supplemented with appropriate amounts of ulvan in *Solea senegalensis* (66), *Chanos chanos* (67), *Labeo rohita* (68), and *Cyprinus carpio* (69), further confirming the *in vivo* immunonutritional functions of ulvan.

The above molecular-weight-dependent functional divergence can be further summarized as a key variable in the four-tier framework for ulvan: the molecular-weight gradient drives pathway preference switching through altered receptor binding modes. Low-molecular-weight (<30 kDa) highly sulfated fragments tend toward a TLR4/TLR2/NF-κB/MAPK-dominated pro-inflammatory phenotype (56, 70), whereas medium-to-low-molecular-weight (~13.5 kDa), uronic-acid-rich fragments favor cGAS-STING-mediated antiviral immunity (9), with the two forming complementary effector phenotypes, as shown in **Table 2**.

**Table 2:** Comparison of immune effector phenotypes of ulvan fractions with different molecular weights.

Molecular weight range	Recognition receptors	Signaling pathways	Immune effector phenotype	References
1–5 kDa	TLR2/TLR4	MAPK, NF-κB	Strong pro-inflammatory, M1 polarization	(56)
~3 kDa	TLR4	PI3K/Akt, NF-κB	Pro-inflammatory (intestinal epithelium)	(70)
~0.5 kDa (oligosaccharides)	TLR4/NLRs (predicted)	NF-κB, JAK-STAT (MAPK ↓)	Strong pro-inflammatory, immunoadjuvant	(59)
~13.5 kDa	TLR4-independent	STING-TBK1-IRF3, NF-κB	Antiviral + pro-inflammatory	(9)
~40 kDa	Not examined	Not examined	Mixed pro-/anti-inflammatory	(60)
>200 kDa (209 kDa)	Not examined	Not examined	Anti-inflammatory/immune tolerance tendency	(34)

### 3.3 Summary of the four-tier framework for ulvan

In summary, the immunomodulatory mechanism of ulvan can be delineated within its four-tier framework as follows: Structurally, ulvan is characterized by low sulfation and high uronic acid content, occupying a unique chemical space among algal sulfated polysaccharides, with its uronic-acid-rich structure potentially providing a basis for intracellular STING pathway activation. Receptor recognition exhibits a coordinated mode involving both surface and intracellular receptors: low-molecular-weight fragments (<30 kDa) primarily engage in surface recognition via TLR4/TLR2 (56, 70), while medium-to-low-molecular-weight fragments (~13.5 kDa) can enter cells to directly activate the STING pathway (9), although the precise mechanism requires further validation. Signaling pathways display parallel activation of TLR4-NF-κB and STING-TBK1-IRF3, with TBK1 serving as a potential intersection node mediating signal integration, although direct evidence for TRIF pathway involvement remains lacking. Immune effector phenotypes exhibit molecular-weight-dependent differentiation: low molecular weight (<30 kDa) predominantly induces pro-inflammation; medium-to-low molecular weight (~13.5 kDa) favors antiviral plus pro-inflammatory effects; and high molecular weight (>200 kDa) tends toward anti-inflammatory/immune tolerance (34). The distinct molecular-weight fractions thus form functionally complementary profiles.

## 4 Carrageenan: the duality of innate immunomodulation

Carrageenan occupies a unique position in the field of immunomodulation: it is one of the most commonly used inflammatory inducers in immunopharmacological research, while also being a potential candidate for antiviral immunotherapy. This dual role stems from the systematic differences among its three subtypes (κ, ι, and λ) in terms of sulfate group number and gelation propensity, enabling them to cover a complete functional spectrum ranging from localized pro-inflammation to systemic immunomodulation (19).

### 4.1 Differential TLR4-NF-κB activation by κ/ι/λ carrageenans

The three carrageenan subtypes activate the TLR4-MyD88-NF-κB pathway through markedly distinct modes, determined jointly by the number of sulfate groups and their gelation propensity. Studies using primary mouse peritoneal macrophages have demonstrated that carrageenan activates NF-κB and AP-1 signaling pathways via the TLR4 receptor, driving massive release of key pro-inflammatory cytokines such as TNF-α, IL-6, and MCP-1 from macrophages (10, 71, 72). Among the subtypes, λ-carrageenan, possessing the highest sulfate content (approximately 35%) and being fully soluble, exhibits the strongest TLR4 activation capacity across multiple studies; κ-carrageenan, due to gelation, produces a sustained, slow release with longer-lasting but lower-intensity inflammatory signals; ι-carrageenan falls in between, displaying moderate solubility and immunostimulatory activity (19, 73, 74).

Cicinskas et al. (37) compared the effects of high-molecular-weight κ- and λ-carrageenans and their low-molecular-weight degradation products (κ-LMWDPs: 0.7–20 kDa; λ-LMWDPs: 10–170 kDa) on mouse peritoneal macrophage function, finding that all samples dose-dependently inhibited macrophage phagocytic activity. In *in vivo* feeding experiments, λ-carrageenan exhibited greater inhibitory effects (49%) than κ-carrageenan (19.5%). Furthermore, all LMWDPs stimulated macrophage motility *in vivo*, with κ-type showing superior effects over λ-type (37). Ai et al. (75), using the murine microglial BV-2 cell line, found that κ- and ι-carrageenan acid-hydrolyzed oligosaccharides (molecular weight approximately 0.5–4.1 kDa) preconditioned at 125–500 μg/mL significantly reduced LPS-induced TNF-α secretion, exhibiting anti-inflammatory activity. In contrast, high-molecular-weight carrageenan (≥63 kDa) at 250–500 μg/mL significantly enhanced LPS-induced TNF-α levels, exhibiting a pro-inflammatory synergistic effect with LPS. At a low concentration of 125 μg/mL, the order of co-inflammatory potency for pure carrageenans was λ-type > ι-type > κ-type (75).

### 4.2 Carrageenan as an inflammasome activator: double-edged sword effects and molecular weight distinction

The activation of the NLRP3 inflammasome by carrageenan is both a highly recognized immunological feature and the core of its application controversy. Current evidence indicates that carrageenan triggers NLRP3 inflammasome assembly through at least the following pathways: (1) Potassium efflux (carrageenan mediates K^+^ efflux through the Panx1/P2X7 pathway, promoting NLRP3 inflammasome assembly) (10, 76); (2) Lysosomal damage (particulate matter, upon phagocytosis by macrophages, causes disruption of lysosomal membrane integrity and release of cathepsin B, thereby promoting NLRP3 inflammasome activation, which is a general mechanism of NLRP3 inflammasome activation) (76); however, whether carrageenan activates NLRP3 through this pathway awaits experimental validation; (3) Mitochondrial ROS production (carrageenan stimulates ROS production in macrophages) (10), and mitochondrial ROS has been established as a critical upstream signal for NLRP3 inflammasome activation (76); however, direct evidence that carrageenan-induced ROS specifically originates from mitochondria is currently lacking.

Following NLRP3 inflammasome activation, Caspase-1 is autocatalytically activated, which subsequently cleaves pro-IL-1β and pro-IL-18 into their mature active forms (10), while also cleaving Gasdermin D to induce pyroptosis (76). This mechanism renders carrageenan a classic inducer of acute inflammatory models, widely used for screening anti-inflammatory drugs and studying inflammatory pathological mechanisms (10, 72).

When evaluating the safety of carrageenan, a strict distinction must be made between the two molecular weight forms. The current mainstream scientific consensus holds that undegraded high-molecular-weight native carrageenan (>100 kDa) used in food products is generally not absorbed in the intestine and does not possess the same inflammatory toxicity as low-molecular-weight degradation products (77, 78). The core of the controversy lies in whether low-molecular-weight degradation products (<50 kDa) are generated during food processing or *in vivo*, particularly the specific degradation product poligeenan produced under acidic high-temperature conditions (e.g., pH < 2, >80 °C) (78), as well as the cumulative effects of long-term exposure (79). Mechanistically, degraded carrageenan (poligeenan) exerts toxicity through multiple pathways: (i) disruption of intestinal epithelial barrier integrity via downregulation of the tight junction protein ZO-1, leading to increased intestinal permeability (80, 81); (ii) activation of TLR4/NF-κB (80, 82) and NLRP3 inflammasome pathways (83, 84) in intestinal epithelial cells and macrophages, driving sustained pro-inflammatory cytokine production; (iii) induction of gut microbiota dysbiosis, characterized by a reduction in beneficial bacteria (e.g., *Bifidobacterium*) and expansion of pro-inflammatory taxa (e.g., *Bacteroides*, *Desulfovibrio*, *Bilophila*) (80, 81, 84); and (iv) induction of oxidative stress and mitochondrial-mediated apoptosis via ROS generation and GSH depletion (82). Additionally, long-term exposure to degraded carrageenan has been associated with carcinogenic potential in animal models (78). These mechanisms collectively explain why low-molecular-weight carrageenan (<50 kDa), unlike its high-molecular-weight native form, is associated with intestinal inflammation and ulceration in animal models (78, 85); moreover, food-grade CGN has been shown to increase intestinal permeability and promote low-grade inflammation *in vivo* (81). *In vitro* and microbial degradation studies have further elucidated the underlying cellular and enzymatic mechanisms (84, 86, 87). The control standards for the proportion of low-molecular-weight components in food-grade carrageenan vary across different countries and regions. **Table 3** provides a comprehensive comparison of the toxicological profiles of high-versus low-molecular-weight carrageenan. This distinction is crucial for understanding the root of the carrageenan safety controversy.

**Table 3:** Toxicological comparison of high-molecular-weight native carrageenan versus low-molecular-weight degraded carrageenan/poligeenan.

Parameter	High-molecular-weight native carrageenan (>100 kDa)	Low-molecular-weight degraded carrageenan/poligeenan (<50 kDa)	References
Intestinal absorption	Minimal to none	Absorbable to a limited extent	(77, 78, 88)
Intestinal epithelial barrier integrity	Generally preserved	Disrupted (occludin, ZO-1 downregulation)	(39, 71)
TLR4/NF-κB activation	Weak to moderate	Strong, sustained	(10, 72)
NLRP3 inflammasome activation	Minimal	Potent activator	(10, 76)
Gut microbiota effects	Minimal dysbiosis	Significant dysbiosis, pro-inflammatory taxa expansion	(89, 90)
Inflammatory potential	Low (at food-grade concentrations)	High, pro-inflammatory cytokine storm	(10, 77, 78)
Genotoxicity/carcinogenic potential	Not established	Potential promotion via chronic inflammation	(78, 91)
Regulatory status (EU)	Permitted (E407) with <5% <50 kDa	Restricted (poligeenan not permitted)	(79) (EFSA)
Regulatory status (US)	GRAS, no specific MW restriction	Not GRAS; not permitted as food additive	(91)
Primary applications	Food additive, topical formulations	Inflammatory model tool (research only)	(10, 19)

Notable progress has been made in the antiviral application of ι-carrageenan. Fröba et al. (92) demonstrated that ι-carrageenan effectively inhibits the replication of the SARS-CoV-2 original strain and its Alpha, Beta, Gamma, and Delta variants *in vitro*. The mechanism involves electrostatic interactions that coat viral particles, blocking viral entry into host cells rather than acting through immunomodulation (92). This finding suggests that the immunomodulatory and direct antiviral activities of carrageenan may be synergistic, offering a new approach for developing local antiviral formulations, such as carrageenan-based nasal sprays.

As shown in **Figure 3**, the immunomodulatory profile of carrageenan displays a dual mode featuring parallel activation of the TLR4-NF-κB pro-inflammatory pathway and the NLRP3 inflammasome pathway. The former ranges from sustained, slow release of κ-type to rapid, potent activation of λ-type as two extremes, while the latter triggers inflammasome assembly through three pathways: K^+^ efflux, lysosomal damage, and mitochondrial ROS production.

**Figure 3:**
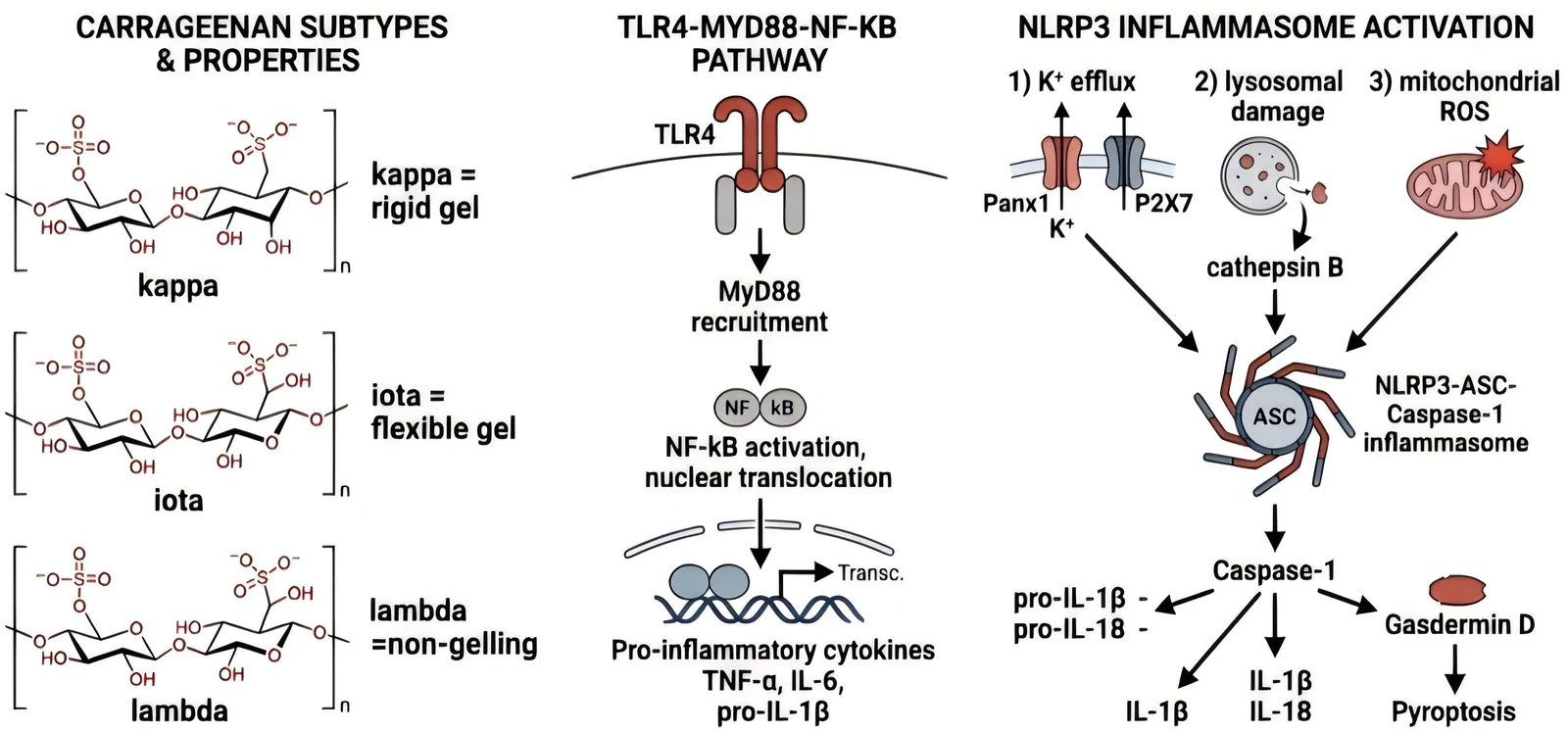
Dual immunomodulatory roles of the three carrageenan subtypes: TLR4 activation and NLRP3 inflammasome activation.

### 4.3 Summary of the four-tier framework for carrageenan

In summary, the dual immunomodulatory nature of carrageenan can be attributed to the following: the degree and gelation behavior jointly regulate the activation modes of the TLR4/NF-κB and NLRP3 inflammasome pathways. Along the TLR4/NF-κB axis, λ-carrageenan, with the highest sulfate content (approximately 35%) and complete solubility, exhibits the strongest pro-inflammatory activation capacity; κ-carrageenan, due to its gelation properties, produces a sustained-release effect with longer-lasting but lower-intensity inflammatory signals; ι-carrageenan falls in between (19, 73, 75). Along the NLRP3 inflammasome axis, carrageenan promotes inflammasome assembly through three pathways: Panx1/P2X7-mediated K^+^ efflux (10), lysosomal damage, and mitochondrial ROS (76), driving IL-1β maturation and release as well as pyroptosis. The parallel activation of these two pathways collectively shapes the tool value of carrageenan in local acute inflammation scenarios, such as inflammatory model construction (72). Within the inverse gradient relationship between sulfate and uronic acid contents among the three polysaccharides, carrageenan occupies an intermediate position in sulfate content and a low position in uronic acid content (19). This structural positioning determines its immunostimulatory profile centered on TLR4/NF-κB and NLRP3 inflammasome activation. Whether carrageenan possesses the ability to activate the cGAS-STING or CLR pathways currently lacks experimental evidence and warrants further investigation. For systemic application, a strict distinction must be made between the toxicological profiles of high-molecular-weight food-grade carrageenan (>100 kDa, not absorbed in the intestine) and low-molecular-weight degradation products (poligeenan, <50 kDa, with intestinal inflammatory and carcinogenic risks) (77–79). **Table 3** provides a detailed comparison of these toxicological profiles.

## 5 Pleiotropic immunomodulatory effects of fucoidan

Among the three polysaccharides, fucoidan has been the most extensively studied in terms of immunomodulation, with its mechanism of action characterized by TLR2/TLR4-mediated recognition and a broad-spectrum regulatory mode involving parallel activation of multiple signaling pathways, including NF-κB and MAPK (7). With TLR4-NF-κB as the main pro-inflammatory pathway (93), NLRP3 inflammasome regulation to counterbalance excessive inflammation (94), and complement C3 activation as an effector amplification mechanism (95), this multi-target network endows fucoidan with application value in immunoadjuvants, anti-inflammation, and antitumor immunity (93, 96).

### 5.1 Dual receptor recognition by TLR4 and CLRs

The immunomodulatory effects of fucoidan are based on its ability to bind multiple pattern recognition receptors, including Toll-like receptors (TLR2/TLR4) and C-type lectin receptors (such as Dectin-1), and to regulate immune responses through multiple signaling pathways, including NF-κB and MAPK (11). Cui et al. (17) prepared six fucoidan fractions with different molecular weights and chemical compositions from Saccharina japonica and found that low-molecular-weight fractions (approximately 5.8–6.2 kDa) with more complex monosaccharide compositions significantly promoted NO, IL-6, TNF-α, and IL-1β secretion by RAW 264.7 macrophages through upregulating the expression of scavenger receptors SR-A and LOX-1 as well as TLR4, indicating that the immunomodulatory activity is closely related to synergistic recognition by multiple receptors (17). Furthermore, fucoidan and Dectin-1 ligands (such as β-glucans) can synergistically enhance the activity of innate immune cells through receptor crosstalk (97).

Li et al. (98) reported a novel mechanism by which fucoidan from the Antarctic brown alga Durvillaea antarctica mediates antiviral effects through the STING pathway. It should be noted that both fucoidan (98) and ulvan (9) can activate type I interferon responses via the STING signaling pathway, but the upstream mechanisms by which they activate STING remain unclear. Based on available evidence, we propose the following working hypothesis: STING phosphorylation induced by fucoidan may depend on reactive oxygen species (ROS)-mediated DNA damage or endoplasmic reticulum stress (ER stress) signaling, rather than direct activation of cGAS. This hypothesis awaits further validation through STING gene knockout or cGAS inhibitor intervention experiments. Elucidating this mechanistic difference is crucial for understanding the distinct roles of the two polysaccharides in antiviral immunity.

Choi et al. (99), through gene set enrichment analysis, found that genes related to the type I IFN signaling pathway were significantly upregulated in mouse bone marrow-derived dendritic cells (BMDCs) following fucoidan treatment, further supporting the hypothesis that fucoidan activates innate immunity through synergistic multi-receptor engagement (99). Kuznetsova et al. (100) compared the immunoadjuvant activities of native fucoidan, highly purified fucoidan, and enzymatic degradation products from Fucus evanescens, finding that all three activated innate and adaptive immune cells and enhanced HBs-specific antibody and Th1/Th2-type cytokine production with comparable activities. Among these, the enzymatic degradation product, despite having a lower molecular weight (50.8 kDa) and a more regular structure (sulfation predominantly at C2, acetylation at C4), exhibited immunostimulatory effects equivalent to the native product, suggesting its potential as a safe candidate vaccine adjuvant (100).

### 5.2 NLRP3 inflammasome regulation and immune balance

Fucoidan exerts a clear inhibitory effect on the NLRP3 inflammasome. Current studies indicate that fucoidan can inhibit NLRP3 inflammasome activation and subsequent IL-1β maturation and secretion through multiple mechanisms, including upregulation of SIRT1 expression (101), enhancement of p62/SQSTM1-dependent selective autophagy (102), and suppression of NF-κB signaling (94). The NLRP3 inhibitory efficacy of fucoidan is influenced by its molecular weight, sulfation degree (103), and source (104), with fucoidans of different structural characteristics exhibiting differential inhibitory activities against NLRP3. This multi-target, multi-pathway NLRP3 inhibition pattern may represent a molecular basis by which fucoidan exerts immunomodulatory effects while avoiding excessive inflammatory responses.

### 5.3 Complement system activation and immunoadjuvant potential

Fucoidan activates complement C3 through the alternative pathway, serving as a key mechanism bridging innate and adaptive immunity. Kuznetsova et al. (100), by comparing the immunoadjuvant activities of native fucoidan, highly purified fucoidan, and enzymatic degradation products from *Fucus evanescens*, found that all three significantly promoted dendritic cell maturation (upregulating CD83, CD80, CD86, and MHCII), enhanced neutrophil adhesion and migration, activated monocytes and enhanced their antigen-presenting function, and increased the cytotoxic potential of NK cells. In a mouse model immunized with hepatitis B surface antigen (HBs-AG), all three fucoidan preparations significantly enhanced total IgG antibody production and Th1 (IFN-γ, TNF-α) and Th2 (IL-4) type cytokine responses, with comparable activities among the three, suggesting that fucoidan may serve as a potential vaccine adjuvant candidate (100). Multiple studies have demonstrated that fucoidan and its derivatives possess the potential to promote hematopoietic recovery in cyclophosphamide-induced immunosuppression models. Anisimova et al. (105) found that chemically modified linear sulfated fucoidan (M-Fuc) alone did not exhibit significant hematopoietic recovery effects, but its related synthetic oligosaccharide (sulfated octasaccharide OS) significantly promoted the recovery of neutrophils, erythrocytes, and platelets, with activity superior to recombinant G-CSF. Li et al. (106) extracted fucoidan (Fuc^hp^) from the sea cucumber *Holothuria polii* and found that it also significantly restored white blood cell and neutrophil counts, with efficacy superior to rhG-CSF and potential advantages in reducing thrombosis risk. Ma et al. (107) further confirmed that simulated digestion products of fucoidan from *Sargassum fusiforme* (DSFF) restored white blood cell, neutrophil, and platelet counts in immunosuppressed mice and promoted bone marrow cell proliferation and differentiation. These findings suggest that fucoidan and its specific oligosaccharides or derivatives may serve as candidate immunorestorative agents for chemotherapy adjunct therapy.

**Table 4** summarizes the key bottlenecks and corresponding strategies for the immunomodulatory application of fucoidan. Additionally, **Table 5** provides a quantitative summary of the bidirectional regulation of the NLRP3 inflammasome by fucoidan as a function of concentration and molecular weight, highlighting its “immune balance” characteristic that distinguishes it from the single activation mode of carrageenan.

**Table 4:** Key bottlenecks and corresponding strategies for the immunomodulatory application of fucoidan.

Challenge	Specific manifestation	Current strategy	Future direction
Structural diversity	High variability in activity among fucoidans from different sources	Standardized extraction and characterization	Establish structure-activity databases
Bidirectional NLRP3 regulation	Concentration-dependent activation/inhibition	Optimize dosage window	Precise elucidation of molecular mechanisms
Safety of complement activation	Overactivation may lead to inflammation	Control sulfate content	Design controllably activated fragments
*In vivo* bioavailability	Low oral absorption rate	Nano-delivery systems	Targeted delivery (108) and controlled release

**Table 5:** Inhibitory mechanisms of fucoidan on the NLRP3 inflammasome (multi-target, multi-pathway).

Concentration/molecular weight	Regulatory direction	Mechanism of action	Key molecules/pathways	Immune effects	References
60 mg/kg (mouse, i.p.); 300 μg/mL (THP-1 macrophages)	Inhibition	Enhances p62/SQSTM1-dependent selective autophagy, promoting NLRP3 inflammasome degradation	p62/SQSTM1, LC3-II/LC3-I, ATG5	Inhibits IL-1β maturation and secretion, attenuates atherosclerotic inflammation	(102)
300 mg/kg (mouse, i.g.); 60 μg/mL (retinal vascular endothelial cells)	Inhibition	Upregulates SIRT1 expression, negatively regulates NLRP3 inflammasome activation	SIRT1, NF-κB	Reduces NLRP3, IL-1β, and IL-1β p17 levels, alleviates hypertensive retinopathy	(101)
25 μg/mL (HK-2 renal tubular epithelial cells)	Inhibition	Suppresses NF-κB signaling pathway, blocks NLRP3-mediated pyroptosis	NF-κB (p65 phosphorylation), GSDMD	Inhibits NLRP3, ASC, Caspase-1, IL-18 expression, protects renal tubular epithelial cells (only this single concentration validated)	(94)
8–25 kDa (low molecular weight); 25–100 μg/mL (RAW 264.7 macrophages)	Inhibition	Inhibits NF-κB/MAPK signaling pathways, blocks NLRP3 inflammasome assembly	NF-κB, MAPK (p38/ERK/JNK), NLRP3, ASC, Caspase-1	Inhibits IL-1β and IL-18 release, attenuates LPS-induced macrophage inflammation	(109)
25, 50 mg/kg (mouse, i.g.)	Inhibition	Inhibits NLRP3-mediated pyroptosis, alleviates oxidative stress	NLRP3, Caspase-1, Caspase-11, GSDMD, TLR4	Dose-dependently reduces IL-1β and IL-18, protects gastric mucosa from ethanol-induced ulcer injury (50 mg superior to 25 mg)	(110)
100 mg/kg (rat, i.g.); 20 μg/mL (mouse podocytes)	Inhibition	Regulates AMPK/mTORC1/NLRP3 signaling axis, inhibits NLRP3 inflammasome-mediated podocyte pyroptosis	AMPK, mTORC1 (raptor), NLRP3	Attenuates renal fibrosis and inflammatory podocyte injury in diabetic kidney disease	(111)

As shown in **Figure 4**, fucoidan initiates innate immune signaling through dual TLR4/CLR receptor recognition, synergistically amplifying pro-inflammatory cytokine secretion via the MyD88-NF-κB and Syk-CARD9 pathways. Concurrently, it maintains immune homeostasis through concentration-dependent bidirectional regulation of the NLRP3 inflammasome and bridges adaptive immune responses via complement C3 activation. This multi-target network endows fucoidan with unique pleiotropic properties in immunomodulation.

**Figure 4:**
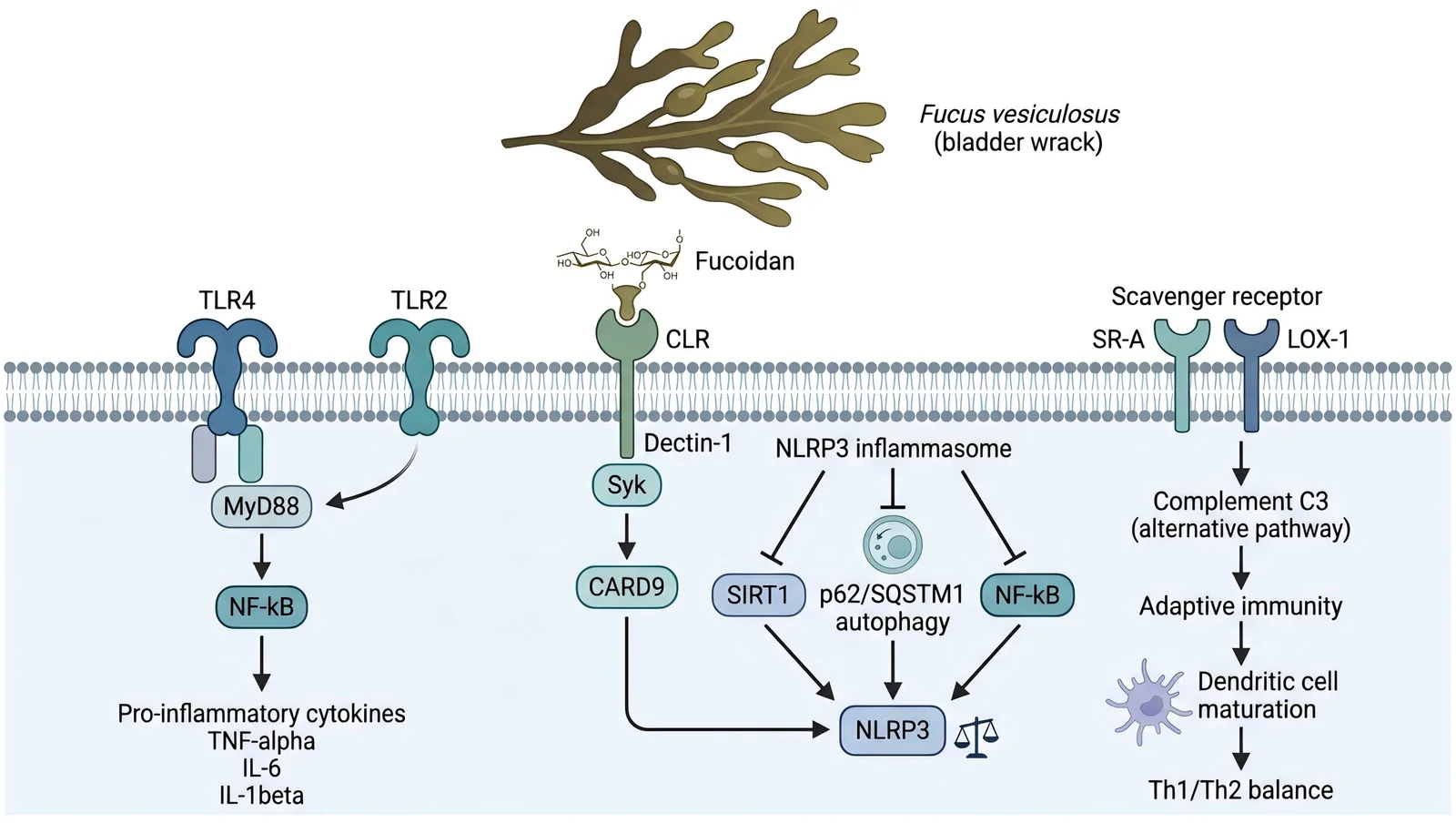
Multi-pathway immunomodulatory mechanisms of fucoidan.

### 5.4 Summary of the four-tier framework for fucoidan

In summary, the pleiotropic immunomodulatory effects of fucoidan can be summarized within its four-tier framework as follows: Structurally, fucoidan is characterized by high sulfation and structural diversity, occupying the highest sulfate content and the lowest uronic acid content among the three algal sulfated polysaccharides (102, 109, 110). Receptor recognition features TLR4 as the core receptor, involving synergistic engagement of multiple receptors including TLR2, scavenger receptors (SRs), and C-type lectin receptors (CLECs) (11, 97). Signaling pathways exhibit a multi-layered network: the TLR4-NF-κB/MAPK axis regulates inflammatory cytokine expression (109); p62/SQSTM1-dependent selective autophagy promotes NLRP3 inflammasome degradation (102); the SIRT1/NF-κB pathway negatively regulates NLRP3 activation (101); the AMPK/mTORC1/NLRP3 axis inhibits podocyte pyroptosis (111); and the NF-κB/NLRP3/GSDMD pathway blocks pyroptosis (94, 110). Immune effector phenotypes achieve Th1/Th2-balanced immune enhancement, with application value in immunoadjuvants, anti-inflammation, and antitumor immunity.

## 6 Systematic comparison of immune signaling pathways and translational scenario projection for the three polysaccharides

Building upon the in-depth dissection of each polysaccharide’s mechanisms in the preceding three sections, this section undertakes a systematic horizontal comparison across four signaling pathways (TLR4, NLRP3, Dectin-1/CLR, and cGAS-STING) to delineate a “pathway preference-immune effector-glycan structure” triangular complementary map among the three polysaccharides (based on theoretical extrapolation from existing literature, pending further experimental validation). Subsequently, differentiated application strategies are projected for three clinical scenarios: viral infection, tumor immunity, and allergic diseases.

### 6.1 Differential activation profiles of TLR/NLR/CLR/cGAS-STING pathways

The recognition and regulatory patterns of the three polysaccharides toward the four key pathways (TLR4, NLRP3 inflammasome, Dectin-1/CLR, and cGAS-STING) exhibit clear gradient differences. Ulvan features the cGAS-STING pathway as its core, inducing type I interferon responses through the STING-TBK1-IRF3 axis (9). Theoretically, TBK1, as a common downstream node of both the TLR4-TRIF and cGAS-STING pathways, may mediate the integration and amplification of signals from these two pathways (immunological theoretical extrapolation, pending experimental validation). Carrageenan (especially the κ subtype) primarily triggers two distinct downstream branches via TLR4: the first provides a transcriptional priming signal (Signal 1) through the TLR4/TRIF/Syk/ROS non-canonical pathway, driving *Il1b* mRNA expression and pro-IL-1β synthesis, rather than relying on the classical MyD88/NF-κB pathway; the second activates the NLRP3 inflammasome through the Pannexin-1/P2X7/K^+^ efflux mechanism, mediating Caspase-1 activation and mature IL-1β secretion (Signal 2). The two pathways operate in concert to form a signal amplification cascade, inducing robust inflammatory responses (10). Fucoidan displays the most diverse receptor recognition profile, involving synergistic engagement of TLR4, Dectin-1 (CLR), and scavenger receptors (SRs) (17, 97). At the NLRP3 inflammasome level, fucoidan exerts inhibitory effects through multiple pathways, including p62/SQSTM1-dependent selective autophagy (102), SIRT1-mediated negative regulation (101), and NF-κB signaling suppression (94, 110).

The sulfate and uronic acid contents of the three polysaccharides exhibit an inverse gradient relationship: sulfate content ranks as fucoidan > carrageenan > ulvan, whereas uronic acid content follows the opposite order, with ulvan being significantly higher than carrageenan and fucoidan (both of which generally have low levels) (2). These structural differences may influence their PRR binding preferences and signaling pathway selectivity. High sulfate density may enhance electrostatic interactions between polysaccharides and the TLR4/MD-2 complex (112), thereby affecting TLR4 signaling intensity; however, the specific direction of the effect (activation or inhibition) depends on the polysaccharide source and structural characteristics (50). The high uronic acid content of ulvan may be associated with its cellular internalization capacity and cGAS-STING pathway activation (9); however, the causal mechanism linking uronic acid content to STING activation awaits direct experimental validation. Furthermore, the molecular weight of carrageenan significantly influences its immunological activity (113), and κ-carrageenan can activate the NLRP3 inflammasome through the Pannexin-1/P2X7/K^+^ efflux mechanism (10). Overall, the causal chain between structure and pathway preference outlined above still requires systematic validation through direct experimentation.

### 6.2 Comparison of immune effector phenotypes and translational scenario projection

The immune effector phenotypes of the three polysaccharides cover a full spectrum from “strong pro-inflammation” through “antiviral immunity” to “balanced immune enhancement,” directly corresponding to distinct clinical translation scenarios. The immune effects of ulvan are characterized by type I IFN production as a hallmark output, activating the host’s innate antiviral immunity through the cGAS-STING pathway (9). This pathway is triggered upon viral entry into cells, suggesting that ulvan may intervene in immune defense at the early stage of viral infection. Carrageenan exhibits the broadest effector spectrum, ranging from κ-type-induced intense local inflammation (suitable for acute inflammatory models and local immunostimulation), through ι-type antiviral activity (suitable for local antiviral formulations), to λ-type systemic immunostimulation (suitable for vaccine adjuvants). However, the risk of NLRP3 inflammasome hyperactivation limits its systemic application. Currently available evidence, albeit limited, suggests that high-molecular-weight carrageenan (>100 kDa) does not induce obvious systemic toxicity or hematological abnormalities under short-term, low-dose exposure (37, 114). However, HMW carrageenan itself exerts immunomodulatory effects, including inhibition of macrophage phagocytic activity, and the safety differences between HMW carrageenan and its low-molecular-weight degradation products have not been systematically validated through dose-response studies or long-term exposure experiments. Consequently, the “safety window” for HMW carrageenan remains toxicologically undefined, warranting larger-scale, longer-term human studies and systematic toxicological evaluation. Kuznetsova et al. (100) demonstrated that fucoidan activates innate immune cells and promotes Th1/Th2-balanced immune responses. Additionally, cumulative evidence indicates that fucoidan negatively regulates the NLRP3 inflammasome through multiple pathways (94, 101, 102, 110), suggesting its potential value in regulating inflammatory responses; however, its specific regulatory role in the tumor immune microenvironment and the mechanisms involving complement C3 warrant further investigation.

In projecting translational scenarios, a clinical-need-based decision framework can be proposed. In early-stage viral infection, ulvan activates the cGAS-STING pathway to enhance type I IFN responses for inhibiting viral replication, while fucoidan enhances adaptive antibody responses through complement C3 activation for sustained protection. The combination may achieve a dual effect of “acute antiviral + long-term immune memory.” Mechanistically, this synergy has immunological plausibility: type I IFN (dominated by ulvan) promotes cross-presentation by dendritic cells, while complement C3d (dominated by fucoidan) lowers the B-cell activation threshold; together, they may synergistically enhance the breadth and persistence of antigen-specific immune responses (awaiting further experimental validation). In tumor immunity, λ-carrageenan recruits M1 macrophages and dendritic cells and increases activated T lymphocyte infiltration into tumor tissues (115), while fucoidan activates dendritic cells through the TLR4 pathway (93) and exerts antitumor effects by targeting C-type lectin receptors (11, 116), suggesting that both classes of polysaccharides hold application potential in antitumor immunomodulation. In allergic disease scenarios, the bidirectional NLRP3 regulation and Th1/Th2 balancing capacity of fucoidan may help correct Th2-biased immune dysregulation, while ulvan’s cGAS-STING pathway activation may suppress Th2-type inflammation through type I IFN signaling (theoretical projection). This synergistic immunomodulatory strategy represents a promising direction for future research. However, special attention must be paid to the molecular weight selection of carrageenan: only high-molecular-weight native carrageenan (>100 kDa) is suitable for such synergistic applications. Furthermore, the dose ratio and administration timing for combination therapy require experimental optimization. Based on theoretical projection of immune response kinetics, ulvan exerts its cGAS-STING antiviral effects during the early phase of viral infection (0–48 hours), whereas fucoidan’s complement activation may enhance adaptive immunity at a later phase (>72 hours). This temporal mismatch could be exploited for optimized synergy, while avoiding the possibility that fucoidan’s anti-inflammatory effects might diminish the pro-inflammatory environment required for ulvan activity.

As shown in **Figure 5**, the three polysaccharides display clear gradient differences in their PRR pathway activation profiles: ulvan is characterized by dual TLR4/cGAS-STING pathways, carrageenan centers on dual TLR4/NLRP3 pathways, and fucoidan covers three pathways (TLR4/CLR/NLRP3). Together, they form a gradient spectrum from “dual pathways” to “multi-pathway network.” This difference can be attributed to the inverse gradient relationship between sulfate and uronic acid contents (sulfate content: fucoidan > carrageenan > ulvan; uronic acid content: ulvan > carrageenan ≈ fucoidan), providing structural guidelines for rational polysaccharide design based on target immune pathways.

**Figure 5:**
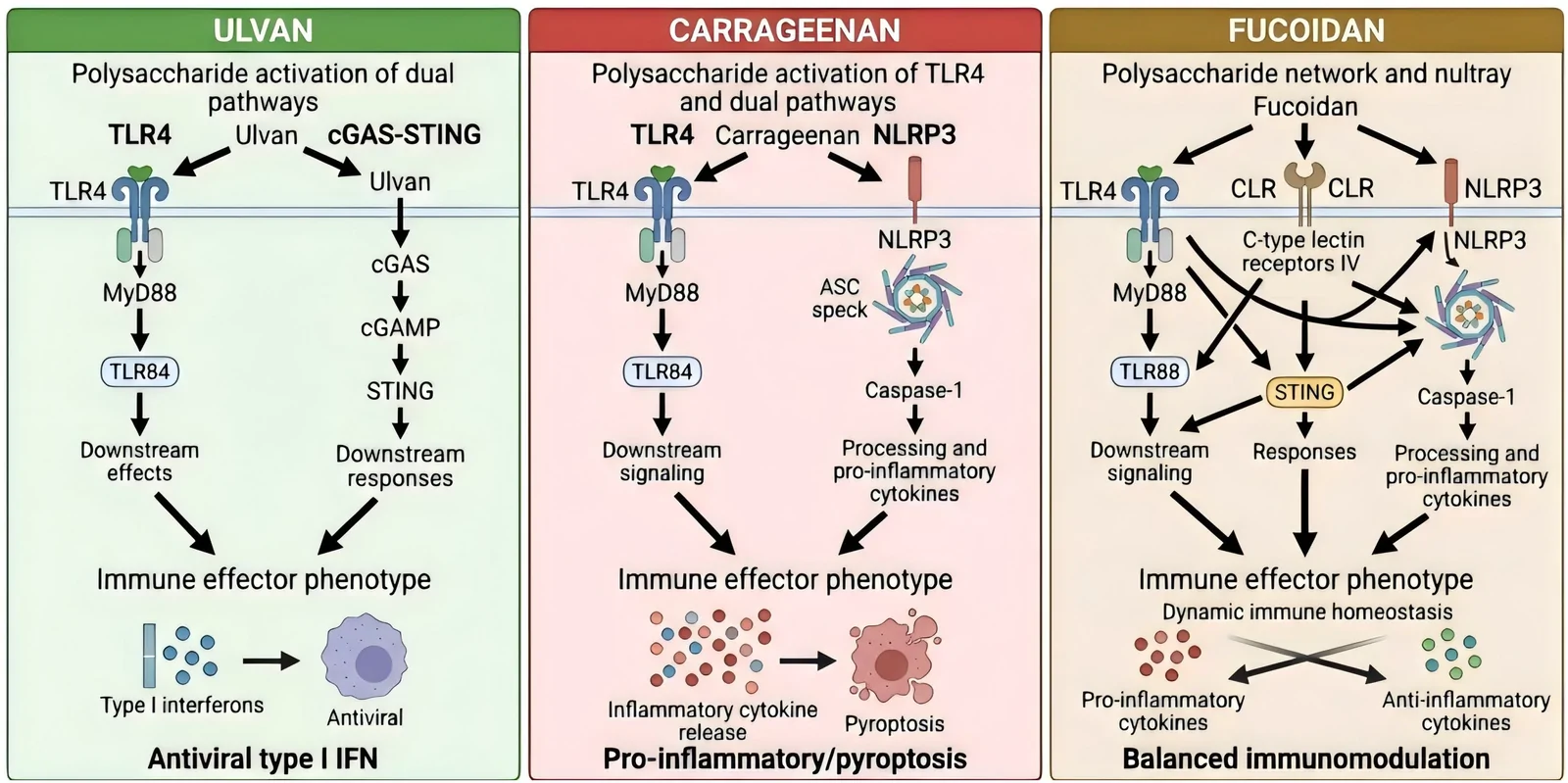
PRR signaling pathway activation profiles and immune effector comparison of the three polysaccharides.

## 7 Research gaps, controversies, and translational perspectives

Despite significant progress in understanding the innate immunomodulatory mechanisms of the three algal sulfated polysaccharides, the translation from basic research to clinical application still faces challenges, including insufficient structural characterization precision and inconsistencies between *in vitro* and *in vivo* results (1). Furthermore, marked differences exist in safety profiles among polysaccharides of different molecular weights and sources (37). Therefore, translational research in this field still requires systematic structure-activity-safety relationship analyses. Advances in precise structural elucidation, establishment of standardized *in vivo* immune evaluation systems, and systematic exploration of the synergistic immunomodulatory potential of the three polysaccharides represent key directions for overcoming these translational bottlenecks.

### 7.1 Challenges in refining structure-activity relationships

The widespread use of crude extracts is the primary source of poor reproducibility in current structure-activity relationship studies. The vast majority of immunomodulatory studies employ hot water-extracted crude polysaccharides or simply fractionated polysaccharide mixtures, and the effects of precise sulfate positions and glycosidic linkage configurations on receptor binding have not been elucidated at the molecular level (15). NMR techniques face severe peak overlap issues in distinguishing sulfate positional isomers, particularly in fucoidan, a highly sulfated and structurally complex polysaccharide, where the precise ratios of C-2 and C-4 sulfate substitutions are often difficult to determine (117). Ion mobility mass spectrometry and two-dimensional NMR-based methods for discriminating sulfate positional isomers are prerequisites for establishing precise structure-activity relationships.

Precise synthesis of oligosaccharide fragments is the key to overcoming this bottleneck. Through chemical or enzymatic synthesis of oligosaccharide fragments with defined sulfate positions and degrees of polymerization, the contributions of individual structural parameters (such as sulfate position, glycosidic linkage configuration, and degree of polymerization) to receptor binding and immunological activity can be systematically assessed, thereby enabling the establishment of precise structure-activity relationship models (118). However, due to the much greater synthetic difficulty of algal sulfated polysaccharides compared to neutral polysaccharides, chemical synthesis of ulvan and fucoidan oligosaccharides is still in its infancy.

### 7.2 *In vitro*-*in vivo* discrepancies and translational pathways

The inconsistency between *in vitro* cellular experiments and *in vivo* animal model results represents another core challenge in the field. The poor *in vivo* bioavailability of polysaccharides is a major contributor to the *in vitro-in vivo* discrepancy. As high-molecular-weight polyanions, sulfated polysaccharides are barely absorbed through the gastrointestinal tract after oral administration (119). Their *in vivo* immunomodulatory effects may be partially dependent on short-chain fatty acids and oligosaccharide fragments generated through gut microbiota metabolism. In addition, although some studies have made preliminary observations on the oral absorption and tissue distribution of fucoidan (120), Zhong et al. (121), in a systematic review, still identified pharmacokinetics as a direction requiring urgent summarization and validation. This underscores that critical parameters such as intestinal mucosal barrier permeability, plasma protein binding, and tissue distribution of polysaccharides remain severely understudied, and the correspondence between effective *in vitro* concentrations and actual *in vivo* exposures is difficult to establish. The development of standardized *in vivo* immune evaluation systems (including unified administration routes, dosage ranges, and immunological endpoint indicators) would help bridge the gap between *in vitro* and *in vivo* results, improving comparability and reproducibility across studies.

Nanodelivery systems represent a key translational strategy for addressing the *in vivo* bioavailability issue of polysaccharides. Carriers such as liposomes and chitosan nanoparticles have been employed to encapsulate fucoidan to enhance its mucosal absorption and stability (122). Liposomes have also been used to encapsulate ulvan to improve its formulation stability and antibacterial activity (123). Tsou et al. (108) utilized diatom-derived mesoporous silica nanoparticles loaded with fucoidan to achieve chemo-photodynamic combination therapy, suggesting that nanotechnology may provide a new platform for polysaccharide-based drug delivery. However, nanocarriers themselves may induce immune-related adverse effects such as complement activation and cytokine release, and their safety assessment must be considered synergistically with the polysaccharides themselves.

### 7.3 The roots of the carrageenan safety controversy

Among the three polysaccharides, the safety controversy surrounding carrageenan is the most prominent, rooted in the fundamental contradiction between its two identities as an “inflammatory model tool” and a “candidate immunotherapeutic molecule.” The safety of carrageenan as a food additive remains controversial (89, 124, 125). Animal studies have shown that long-term consumption of carrageenan-containing diets can disrupt gut microbiota homeostasis, increase intestinal permeability, and induce inflammatory responses (90), suggesting that the effects of chronic low-dose exposure on intestinal health warrant continued attention. Low-molecular-weight carrageenan (<50 kDa) is a commonly used inflammatory stimulus that induces macrophages to produce pro-inflammatory cytokines such as TNF and IL-1β through activation of the TLR4/NF-κB pathway and the NLRP3 inflammasome (10), and triggers intestinal inflammatory responses in animal models (71). Song et al. (88) demonstrated that low-molecular-weight carrageenan, following oral administration, can enter the mouse liver and kidney, indicating a certain degree of *in vivo* absorption. Furthermore, control standards for the proportion of low-molecular-weight components in food-grade carrageenan vary across countries and regions. The European Union stipulates that components below 50 kDa shall not exceed 5% (79), while the FDA has not imposed specific restrictions (91). This regulatory divergence reflects ongoing scientific uncertainty regarding the threshold of low-molecular-weight carrageenan that constitutes a safety risk. In contrast, the least safety data are available for ulvan, while fucoidan benefits from nearly two decades of clinical use in kidney disease, providing a relatively larger body of safety data (11). **Table 3** summarizes the key toxicological distinctions between high-molecular-weight and low-molecular-weight carrageenan.

### 7.4 Translational landscape and clinical perspectives

The translation of algal sulfated polysaccharides from bench to bedside requires a clear understanding of their clinical development status, regulatory classification, and remaining challenges. This section provides a concise overview of these translational aspects across the three polysaccharide classes.

#### 7.4.1 Clinical trials and human studies

Fucoidan has the most extensive clinical experience among the three polysaccharides. Clinical trials have explored its applications in chronic kidney disease, cancer supportive care, and inflammatory joint conditions (11, 96, 105). However, many of these trials are small-scale, open-label, or non-randomized, and larger placebo-controlled trials are needed to establish definitive efficacy.

Carrageenan has been evaluated primarily for topical antiviral applications. Notably, ι-carrageenan-based nasal sprays have undergone clinical evaluation for prevention and treatment of common cold and COVID-19, with some studies demonstrating reduced viral load and symptom severity (92). Carrageenan-containing lozenges and oral rinses have also been studied for their local immunomodulatory effects. However, systemic carrageenan administration has not advanced to clinical trials due to safety concerns discussed in Section 7.3.

Ulvan currently has no registered clinical trials, reflecting its earlier stage of preclinical development. The recent discovery of its cGAS-STING pathway activation (9) may catalyze interest in ulvan as a vaccine adjuvant or antiviral immunomodulator, but extensive toxicology and pharmacokinetic studies are prerequisites for clinical advancement.

#### 7.4.2 Regulatory classification and quality considerations

From a regulatory perspective, the three polysaccharides occupy different categories:

Carrageenan is the most established from a regulatory standpoint, with approvals as a food additive (E407/E407a in the EU, GRAS in the US) and as an excipient in pharmaceutical formulations (77, 79). However, its dual identity as both a food ingredient and an inflammatory tool creates regulatory complexity: the permitted use levels and molecular weight specifications differ between food and pharmaceutical applications.

Fucoidan is generally marketed as a dietary supplement in many countries, with a less defined regulatory pathway for pharmaceutical development (126–130). Clinical development would require regulatory approval aligned with the intended therapeutic indication.

Ulvan remains in the preclinical research domain without established regulatory precedent for food or pharmaceutical use. Any path forward would require comprehensive safety and toxicology packages aligned with regulatory expectations.

Critical quality attributes for regulatory acceptance across all three polysaccharides include: (i) well-characterized molecular weight distribution and consistency across batches; (ii) defined sulfate content and substitution pattern; (iii) purity criteria (protein, endotoxin, heavy metal limits); and (iv) stability data demonstrating consistent bioactivity over shelf life.

#### 7.4.3 Remaining challenges for clinical translation

Several cross-cutting challenges must be addressed to advance these polysaccharides toward clinical use:

1. Dose optimization is critical because the magnitude of the immunological effects observed for both fucoidan (NLRP3 suppression) and carrageenan (NLRP3 activation) is strongly concentration-dependent. Furthermore, while fucoidan demonstrates a favorable safety profile, the pro-inflammatory and tissue-damaging effects of carrageenan highlight the necessity of careful dose selection to avoid potential adverse outcomes in therapeutic applications (10, 102, 110).
2. Administration route: Oral bioavailability remains poor for all three polysaccharides due to their high molecular weight and polyanionic nature (106, 131–134). Local administration (topical, nasal, inhalation) is more feasible for immediate applications; systemic delivery requires nanocarrier or chemical modification strategies (85, 122, 135–137).
3. Safety endpoints: Long-term safety studies are lacking for ulvan and fucoidan (beyond existing food/supplement use) (138–140). For carrageenan, the unresolved safety controversy regarding low-molecular-weight fractions continues to hamper broader clinical acceptance (Section 7.3; 79).
4. Combination strategies: As proposed in Section 6.2, the complementary mechanisms of the three polysaccharides suggest potential for synergistic combinations, but pharmacokinetic and pharmacodynamic interactions require systematic study.

## 8 Conclusion and outlook

The differential innate immune modulation by the three algal sulfated polysaccharides can be attributed to receptor preference and pathway selection driven by an inverse gradient relationship between sulfate and uronic acid contents. Sulfate content ranks as fucoidan > carrageenan > ulvan, whereas uronic acid content follows the opposite trend.

The theoretical innovations of this review are twofold. First, we have established a unified four-tier analytical framework (structural features → receptor recognition → signaling pathways → immune outcomes) that enables, for the first time, a systematic horizontal comparison of three algal sulfated polysaccharides from distinct taxonomic divisions. Second, we have identified and formalized the inverse gradient relationship between sulfate and uronic acid contents as a core structure-activity principle that provides a unified structural explanation for the observed differences in receptor preference and pathway selection across polysaccharide types. The key mechanistic and translational conclusions are as follows:

- Fucoidan, with the highest sulfation (5%–38%) and low uronic acid levels, exerts pleiotropic immunomodulatory effects through a TLR4/CLR/NLRP3 multi-pathway network, showing the greatest promise as an immunoadjuvant and in antitumor immunotherapy.
- Carrageenan, with intermediate sulfation (15%–35%) and negligible uronic acid, relies on dual TLR4/NLRP3 pathways to cover a functional spectrum from localized pro-inflammation to systemic immunostimulation. Its role remains more as a “tool” than a “drug”; only high-molecular-weight native carrageenan (>100 kDa) is suitable for topical application, whereas low-molecular-weight degradation products (<50 kDa) must be strictly avoided due to systemic toxicity risks.
- Ulvan, with the lowest sulfation (8%–15%) and the highest uronic acid content (6.5%–19.0%), exhibits a distinctive advantage in antiviral immunity through coordinated TLR4/cGAS-STING dual pathways. Its STING activation may be linked to the regulation of the cytosolic ionic microenvironment by uronic acid.

These findings provide structural guidelines for rational polysaccharide design: enhancing TLR4/NF-κB activation should prioritize high sulfate content; targeting the cGAS-STING antiviral pathway should retain the uronic acid backbone; and avoiding NLRP3 hyperactivation requires controlled molecular weight (30–100 kDa) and moderate sulfation. In translational applications, highly sulfated fucoidan is suitable for systemic immunoadjuvants, uronic-rich ulvan for early antiviral intervention, and high-molecular-weight carrageenan for localized intestinal immunomodulation only.

Future research should integrate glycobiology, immunology, and nanotechnology to dynamically monitor the conformational changes and receptor-binding kinetics of polysaccharides within specific immune microenvironments, thereby transforming structural description into functional prediction and advancing algal sulfated polysaccharides as precision immunotherapeutic tools.
